# Ultrasound-Guided Radiofrequency Ablation for Papillary Thyroid Microcarcinoma: Efficacy and Safety in a Cross-Country Multicenter Retrospective Study

**DOI:** 10.1155/ije/1438786

**Published:** 2025-09-27

**Authors:** Wei-Che Lin, Chi-Cheng Chen, Yi-Ju Wu, Ming-Hsun Wu, Kai-Lun Cheng, Wen-Hung Wang, Le Thi My

**Affiliations:** ^1^Department of Diagnostic Radiology, Kaohsiung Chang Gung Memorial Hospital and Chang Gung University College of Medicine, Kaohsiung, Taiwan; ^2^Department of Diagnostic Radiology, Kaohsiung Municipal Fong Shan Hospital–Under the Management of Chang Gung Medical Foundation, Kaohsiung, Taiwan; ^3^Department of Diagnostic Radiology, Kaohsiung Municipal Ta-Tung Hospital, Kaohsiung, Taiwan; ^4^Thyroid Head and Neck Ablation Center, Kaohsiung Chang Gung Memorial Hospital, Kaohsiung, Taiwan; ^5^School of Medicine, College of Medicine, National Sun Yat-Sen University, Kaohsiung, Taiwan; ^6^Department of Surgery, Jen-Ai Hospital, Taichung, Taiwan; ^7^Department of Surgery, National Taiwan University Hospital, Taipei, Taiwan; ^8^Department of Medical Imaging, Chung Shan Medical University Hospital, Taichung, Taiwan; ^9^School of Medicine, Chung Shan Medical University, Taichung, Taiwan; ^10^School of Medical Imaging and Radiological Sciences, Chung Shan Medical University, Taichung, Taiwan; ^11^Department of Otolaryngology, Cathay General Hospital, Taipei, Taiwan; ^12^Department of Otolaryngology, Sijhih Cathay General Hospital, New Taipei City, Taiwan; ^13^School of Medicine, National Tsing Hua University, Hsinchu, Taiwan; ^14^School of Medicine, Fu Jen Catholic University, New Taipei City, Taiwan; ^15^Department of Radiology, Vinmec Healthcare System, Hanoi, Vietnam

**Keywords:** papillary thyroid microcarcinoma (PTMC), radiofrequency ablation (RFA), thyroid, ultrasound (US)-guided

## Abstract

**Objectives:** This multicenter cross-country study aimed to assess and compare the short- and long-term efficacy and safety of ultrasound (US)-guided radiofrequency ablation (RFA) for low-risk papillary thyroid microcarcinoma (PTMC) in distinct national treatment settings.

**Materials and Methods:** This retrospective study analyzed low-risk PTMC patients who received US-guided RFA at 6 medical centers in 2 countries (Taiwan and Vietnam) between November 2017 and April 2023. US and computed tomography (CT) were performed to assess and compare the PTMC tumors, changes in tumor size, and disease progression. Repeated measure ANOVA and two-way mixed ANOVA were used to analyze within-group and between-group differences among volume and volume reduction ratio (VRR). Student's *t*-test and the standard chi-square test were used to compare between-group data, while paired *t*-test was used to compare within-group data.

**Results:** A total of 206 patients (mean age: 44.8 ± 12.5 years [range, 19–87], 44 men) with 224 PTMC tumors were enrolled and treated. Four (1.9%) patients reported transient hoarseness as complications. All tumors exhibited a reduction in size (*p* < 0.001) at 12 month post-RFA, while 58.5% (131/224) of PTMCs had completely disappeared under US examination at the last follow-up. One patient had developed ipsilateral cervical LN metastasis at the 6-month follow-up. While the VRR showed a statistical difference between the two countries in the short term (*p* < 0.001), this difference was not observed at 2 year follow-up (*p*=0.159).

**Conclusions:** US-guided RFA is an effective and safe procedure for patients with PTMC. Furthermore, we noted no long-term differences in terms of procedural outcomes under the distinct national treatment settings.

## 1. Introduction

Over the last 3 decades, thyroid cancer has emerged as one of the fastest-growing malignancies globally, with papillary thyroid carcinoma (PTC) being the primary contributor to the rapidly increasing number of diagnoses in many parts of the world, including the United States, Taiwan, and Vietnam [1–4]. Papillary thyroid microcarcinoma (PTMC) is a subtype of PTC, with a maximum tumoral diameter of 10 mm or less associated with an excellent prognosis [5] and a reported 10 year disease-specific survival rate of up to 99.5% [6].

According to the 2015 American Thyroid Association (ATA) guidelines [7], the 2017 Korean Society of Thyroid Radiology guidelines [8], and the 2019 European Society for Medical Oncology guidelines [9], hemithyroidectomy is recommended as the first-line treatment for PTMC. However, surgery carries the inherent risk of complications, such as hematoma, injury to the recurrent laryngeal nerve, infection, and surgical scarring [10].

Due to the low-risk course of PTMC, active surveillance has also been adopted as an alternative approach for patients, as it bears no complications and offers a similar outcome to immediate surgery [7–9]. Nevertheless, the uncertainty related to active surveillance may lead to anxiety and a psychological burden in patients, thereby greatly impacting the quality of life [11].

In recent years, the importance of functional preservation in the treatment of thyroid disease has been growing. Thus, several alternative minimally invasive techniques have been proposed for treating PTMC, including ultrasound (US)-guided microwave ablation [12–14], laser ablation [15–17], and radiofrequency ablation (RFA) [18–24]. Indeed, studies on thermal ablation of PTMC have demonstrated excellent clinical outcomes with low complication rates, despite having relatively short follow-up periods. Moreover, in the Korean Society of Thyroid Radiology [8] and European Thyroid Association and Cardiovascular and Interventional Radiological Society of Europe [25] guidelines, RFA is proposed as an alternative treatment option for PTMC patients.

In Asia, several countries including Korea [22, 23], China [18–21], and Taiwan [24] have reported similarly positive results with regards to RFA for PTMC patients. Studies involving multiple centers within single countries have been conducted in Korea [23] and China [13]. However, this is the first retrospective study to include patients from multiple medical centers in different countries, as well as the first study to report on the efficacy and safety of US-guided RFA in PTMC and thyroid carcinoma patients from Vietnam. The objective of this study was to examine and compare the therapeutic efficacy and safety of US-guided RFA for PTMC patients using data from 4 medical centers in Taiwan and 2 medical centers in Vietnam.

## 2. Materials and Methods

### 2.1. Patients

The study protocol was reviewed and approved by the Research Ethics Committee of Chang Gung Memorial Hospital (IRB no. 202301622B0), and it adhered to the Declaration of Helsinki principles and the International Conference on Harmonization for Good Clinical Practice. Signed informed consent was waived as personal patient data were thoroughly deidentified in this retrospective study.

This retrospective study reviewed all patients with pathology-proven PTC who received US-guided RFA at 6 medical centers in Taiwan and Vietnam between November 2017 and April 2022. The baseline data of all patients, including clinical characteristics, nodular parameters, and laboratory results, were collected prior to the RFA treatment from medical records. All patients provided informed consent before each procedure.

### 2.2. Inclusion and Exclusion Criteria

The inclusion criteria for RFA treatment were (1) solitary or multiple PTCs confirmed by US-guided fine-needle aspiration (FNA); (2) no imaging findings indicating evidence of extrathyroidal extension, lymph node metastasis, or distant metastasis on both US and head and neck computed tomography (CT) scans; (3) no abnormal findings of vocal cord paralysis during laryngoscopy examination; (4) no abnormal coagulation function test; and (5) refusal or ineligibility for surgery.

**Figure 1:**
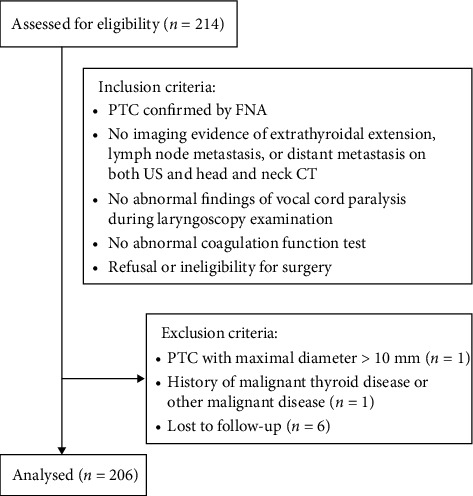
The CONSORT flow diagram of the study. PTC = papillary thyroid carcinoma. FNA = fine needle aspiration.

The exclusion criteria for this study were as follows: (1) PTC with a maximal diameter greater than 10 mm; (2) previous history of malignant thyroid disease or other malignant disease; and (3) incomplete follow-up data (lost to follow-up before 6 months). The CONSORT flow diagram is shown in Figure 1.

### 2.3. Preablation Assessment

CT scans were performed to assess the size and extension of PTMC tumors and to exclude cervical lymph node metastasis or distant metastasis. On the day of the RFA procedure, US was used to record the three orthogonal tumoral diameters, volume, location, and microcalcification. Equation *V* = *πabc*/6 (*V*: volume; *a*: the largest diameter; *b* and *c*: the other two perpendicular diameters) was used to calculate the volume of the PTMC. All tumors underwent cytopathological diagnosis using US-guided FNA.

Prior to RFA, laboratory examinations were conducted, including platelet count, blood coagulation test, serum thyroglobulin, thyroid function test, and thyroglobulin antibody test.

### 2.4. Ablation Technique

The RFA procedures were all performed in an outpatient setting. All patients received a single RFA session regardless of presenting with a singular or multiple PTMCs. The ablation procedures were performed by 5 individual radiologists, each with over 10 years of experience in US-guided procedures.

Prior to the RFA procedure, a mixed solution of local anesthesia (including 2% lidocaine hydrochloride, epinephrine, and sodium bicarbonate) was injected at the puncture site and around the thyroid gland [26]. Hydrodissection with 5% dextrose in water was infused around the thyroid gland to create an insulating zone between the thyroid gland and neighboring critical structures, such as the trachea, nerves, esophagus, and blood vessels, to prevent thermal injury.

The electrode tip size was chosen according to US examination guidelines. The PTMCs were approached via the trans-isthmic route, with the electrode penetrating the thyroid parenchyma and inserted through the deepest and most distant part of the tumors using the moving shot technique [27]. An extratumoral region of more than 2 mm into the adjacent normal thyroid parenchyma was also ablated to establish a sufficient zone to prevent residual tumor presence and tumor recurrence [24]. Each procedure was terminated when the targeted area had transformed into a transient hyperechoic state [26].

### 2.5. Follow-Up Evaluations

After the RFA procedure, all patients were kept under observation in the hospital for at least 30 min to monitor for potential complications. The Society of Interventional Radiology terminology was used to assess major and minor complications [28].

Post-RFA follow-up US evaluations for changes in tumor size, volume reduction ratio (VRR), tumor recurrence, and disease progression, including cervical lymph node and distant metastasis, were performed at 1, 3, 6, 9, and 12 months, and yearly thereafter. The VRR was calculated using the following equation: VRR (%) = initial volume (mL)—final volume (mL) × 100/initial volume. Complete disappearance of the tumor was defined as the treated area becoming only a linear scar or could no longer be measured.

Follow-up contrast-enhanced CT was performed at the 6 month to 1 year post-RFA follow-up to assess the volume of the ablated area and the possibility of tumor recurrence, newly developed cancer, lymph node metastasis or distant metastasis. Regular clinical follow-ups and serum thyroglobulin laboratory examinations were also performed on a regular basis.

If the area treated with RFA was still visible at the 6 month follow-up, a US-guided FNA biopsy would be performed to evaluate the effectiveness of the RFA procedure and to confirm that there was no remaining viable tumor in the treated area. If any unusual imaging results were detected during follow-up, such as a larger treated area that may indicate remaining viable tumor cells, new nodules, or distant metastasis, an FNA would be carried out for further investigation. This applied to all follow-up appointments except for the 1-month follow-up.

### 2.6. Analyses and Statistics

The statistical analyses were performed using SPSS software for Windows (version 26.0, SPSS, Inc., Chicago, IL, USA). Categorical variables were presented as frequencies and percentages, and continuous variables were presented as mean ± standard deviation (SD). Repeated measures the General Lineal Model (GLM) was used to analyze individual-group and between-group (Taiwan versus Vietnam) differences among volume and VRR from baseline to the end of follow-up. Student's *t*-test was used to compare continuous variables between groups, while the standard chi-square test was used for comparison of categorical data. Paired *t*-test was used to compare volume and VRR at an exact time point and its previous time point within groups. A *p* value of < 0.05 was considered statistically significant.

## 3. Results

**Figure 2:**
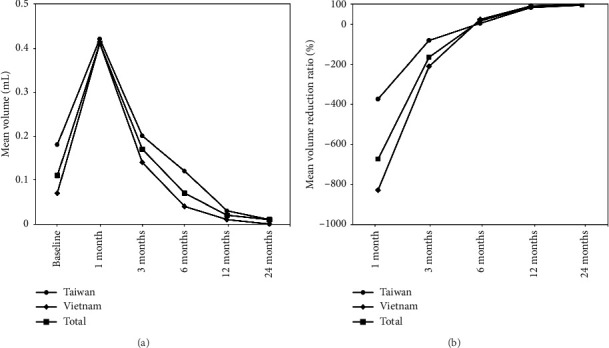
Mean PTMC tumor volume and VRR at each follow-up. (a) The mean tumor volume and (b) the mean VRR of the PTMCs in Taiwan, Vietnam, and both countries before and after RFA at each follow-up. Both countries showed successful treatment responses compared to the baseline, with consistent trends observed in the volume change and VRR between the two countries. PTMC = papillary thyroid microcarcinoma, VRR = volume reduction ratio, RFA = radiofrequency ablation.

The baseline clinical characteristics of the patients and the PTMCs are summarized in Table 1, while the treatment results are shown in Table 2 and Figure 2.

### 3.1. Baseline Clinical Characteristics

The study population consisted of 206 patients with a total of 224 tumors, of which 188 patients had a single tumor and 18 patients had two tumors (Table 1). During the study period, among the 206 patients, 61 (29.6%) had their last follow-up at 6 months, 95 (46.1%) at 12 months, and 50 (24.3%) at 24 months. The average patient age was 44.82 years (ranging from 19 to 71 years), and 78% of the patients were female. Statistically significant differences (*p* < 0.001) were observed in the mean patient age between the two countries, with Taiwan (49.28 ± 13.25 years) having a mean age 6.95 years higher than Vietnam (42.33 ± 11.44 years). In terms of tumor location in the thyroid gland, 80 (35.7%) tumors were located at the left lobe, 124 (55.4%) tumors were located at the right lobe, and 20 (8.9%) tumors were located at the isthmus. There were no significant differences between the two countries in terms of gender distribution (*p*=0.399), number of tumors in each patient (*p*=0.745), or tumor location in the thyroid (*p*=0.090).

### 3.2. Overall Tumor Changes

The mean tumoral size at baseline and at each follow-up is presented in Table 2 and Figure 2(a). The overall mean longest diameter at baseline was 6.26 ± 2.05 mm. The overall mean volume before RFA was 0.11 ± 0.12 mL. An increase in the mean volume to 0.41 ± 0.26 mL was noted at 1 month after RFA, with statistical significance (*p* < 0.001), as compared to the baseline volume. After the transient increase, the mean volume proceeded to decrease to 0.17 ± 0.15, 0.07 ± 0.13, 0.02 ± 0.06, and 0.01 ± 0.03 at 3, 6, 12, and 24 months after RFA, respectively, with the mean volume reaching below the baseline at the 6 month follow-up. All results showed statistical significance (*p* < 0.001) compared to the previous follow-up. A repeated measures ANOVA was performed to compare the effect of time on volume change, which showed a statistically significant effect of time on volume change (F(2.133, 98.102) = 63.257, *p* < 001).

### 3.3. Subgroup Analysis by Country

A comparison between Taiwan and Vietnam showed significant differences in baseline tumor size. The mean longest diameter (Taiwan: 7.44 ± 2.22 mm; Vietnam: 5.61 ± 1.62 mm) and baseline tumor volume (Taiwan: 0.18 ± 0.17 mL; Vietnam: 0.07 ± 0.06 mL) were both significantly larger in Taiwan (*p* < 0.001). Volume remained significantly different at the 6- and 12-month follow-ups (both *p* < 0.001) but not at 1-, 3-, or 24-month follow-ups (*p*=0.370, *p*=0.170, and *p*=0.159, respectively) (Table 2). Both groups showed similar trends of volume reduction, with volumes reaching below baseline by 6 months (Figure 2(a)).

Repeated measures ANOVA confirmed a significant effect of time on tumor volume in both countries (Taiwan: F(2.190, 78.835) = 40.875, *p* < 001; Vietnam: F(1.442, 12.974) = 37.223, *p* < 001). A two-way mixed ANOVA showed a significant main effect of time on volume reduction (F(2.150,96.732) = 49.690, *p* < 001), but no significant effect of country (*p*=0.412) or country-by-time interaction (*p*=0.193).

The mean VRRs at each follow-up are presented in Table 2 and Figure 2(b). The overall mean VRR achieved a positive value at the 6 month follow-up with 16.80 ± 114.07% and reached 96.18 ± 13.29% at the 24 month follow-up. At final follow-up, 127 tumors (56.7%) had completely disappeared. VRR was significantly higher in Vietnam at the 1-, 3-, and 6-month follow-ups (all *p* < 0.001), but not at 12 or 24 months (*p*=0.636 and *p*=0.159, respectively), with both countries showing similar overall trends (Figure 2(b)).

Repeated measures ANOVA confirmed a significant time effect on VRR in both countries (Taiwan: F(1.146, 41.267) = 20.779, *p* < 001; Vietnam: F(1.088, 9.792) = 15.336, *p*=0.003). A two-way mixed ANOVA showed significant main effects of time (F(1.144, 51.480) = 55.840, *p* < 0.001) and country (*F*(1,45) = 20.027, *p* < 0.001), and a significant interaction effect (F(1.144, 51.480) = 20.124, *p* < 0.001), indicating early differences in VRR that diminished over time.

### 3.4. Subgroup Analysis by Center Volume

A post hoc analysis was conducted to compare clinical outcomes between high-volume centers (≥ 50 cases) and low-volume centers (< 50 cases). High-volume centers included Centers No. 1, No. 5, and No. 6, with Centers No. 5 and No. 6 grouped together due to sharing the same operator and clinical protocols. Low-volume centers included Centers No. 2, No. 3, and No. 4. The results are summarized in Table 3.

While low-volume centers demonstrated higher early volume reduction at 6 months and treated slightly larger tumors at baseline (*p*=0.003), the VRR between high- and low-volume centers became comparable by 12 and 24 months (*p*=0.648 and 0.921, respectively). Complete tumor disappearance at 12 and 24 months (*p*=0.775 and 0.834, respectively) and complication rates (*p*=0.374) showed no statistically significant differences.

### 3.5. Complications and Disease Progression

Four patients in Vietnam reported transient hoarseness as complications, all of whom recovered without sequela within 2 months. The patients in this study did not experience any other major complications, such as hematoma, skin burn, nodule rupture, or recurrent laryngeal nerve injuries. All patients maintained normal thyroid function after the RFA treatment.

At the 6 month follow-up, 135 patients (65.9%) underwent repeat US-guided FNA due to persistent hypoechoic appearance or incomplete resolution of the treated nodule on US (Table 1). All repeat FNAs revealed no evidence of malignancy or atypical cells, indicating postablation fibrotic or necrotic changes rather than residual viable tumor. No other patients required re-ablation or surgery during the study period, except one patient described below.

In our study, we noted one case of lymph node metastasis as disease progression in a 26-year-old Taiwanese male who received RFA for a single PTMC tumor located at the right thyroid bed. The original tumor had a longest diameter of 9 mm and a baseline volume of 0.23 mL. At the 6-month follow-up, the tumor size had decreased to 0.08 mL, with a VRR of 65.22%. However, a newly discovered ipsilateral neck lymph node was detected by CT at the 6 month follow-up, which was confirmed as metastatic by US-guided FNA. The patient subsequently underwent additional RFA for the metastatic lymph node, and no evidence of recurrent disease or distant metastasis was observed during continued follow-up through 24 months.

## 4. Discussion

The current study is, to the best of our knowledge, the first international (cross-country) evaluation and comparison of the therapeutic efficacy and clinical safety of RFA for PTMC. Our findings demonstrate that while short-term follow-up results may exhibit differences, no distinction exists with a longer follow-up time in terms of the efficacy of RFA for PTMC patients, regardless of distinct national treatment settings, healthcare system, or equipment operator.

**Figure 3:**
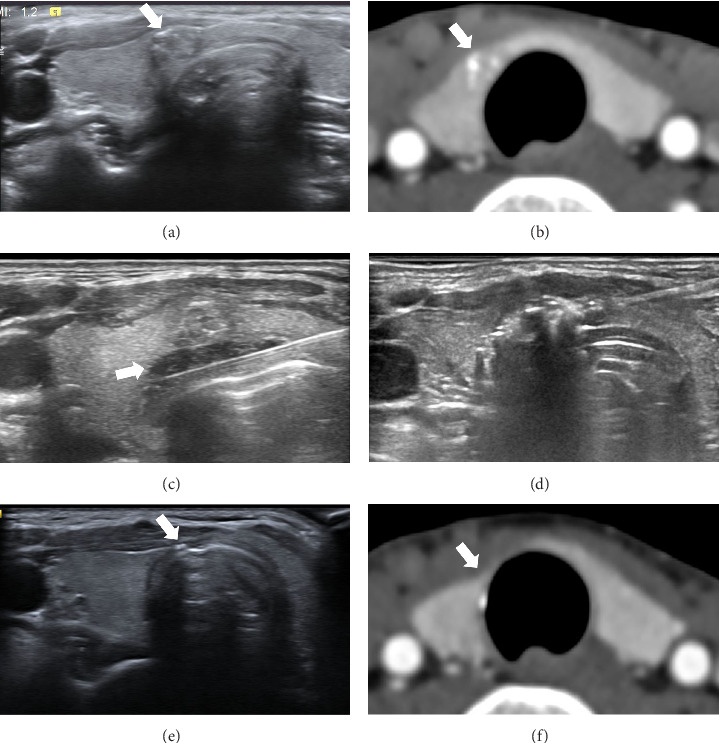
A 46-year-old Taiwanese female with PTMC who underwent RFA. (a) US examination and (b) contrast enhanced CT scan revealed the PTMC (arrows) measuring 7 × 6 × 4 mm in the right isthmus of the thyroid adjacent to the trachea. (c) In the beginning of the RFA procedure, a layer of hydrodissection (arrow) was created to prevent thermal injury to the adjacent trachea. (d) During the RFA procedure, the targeted area was transformed to a transient hyperechoic state. (e) Follow-up US examination at 1 year after RFA showed regression of the PTMC, with a remaining linear scar (arrow) measuring 2 mm and atrophy of the right isthmus of the thyroid. (f) Contrast enhanced CT scan 3 years after RFA showed total regression (arrow) of the PTMC. PTMC = papillary thyroid microcarcinoma, RFA = radiofrequency ablation, CT = CT, US = ultrasound.

The findings of this study indicate that RFA treatment is notably effective in PTMC patients (Figure 3). The mean VRR in both countries exhibited a significant tumor volume reduction at the 12 month follow-up (87.58%) as compared to baseline, with a complete disappearance rate reaching 71.7% at the 12 month follow-up and 99.2% at the 24 month follow-up. Our study also demonstrates the safety of the RFA procedure, with only 4 (1.9%) patients experiencing transient hoarseness and complications; furthermore, patients in the study presented an excellent 1 year prognosis, with only 1 (0.5%) patient presenting with lymph node metastasis at the 6 month follow-up. Previous RFA studies have reported successful VRRs, reaching 54%–99% at the 12 month follow-up, with complication rates ranging from 0% to 3% and a lymph node metastasis rate of 1.4% and 3.4% at the 5 and 10 year follow-ups, respectively [16–20, 29].

Previous studies of the surgical option have reported a 1%–6% recurrence rate [30] and a 1.1%–10.1% complication rate, noting cases of recurrent laryngeal nerve palsy, hypocalcemia, and wound infection [31]. Meanwhile, a propensity-matched cohort study comprised of 884 patients in China also reported RFA to have a shorter procedural time, shorter hospitalization, lower estimated blood loss, and lower complication rate [32] as compared to thyroid lobectomy.

In this study, we observed a statistical difference in the mean baseline tumor volume between Taiwan and Vietnam, which impacted the tumor volume results at each follow-up. More specifically, the tumors reported in Vietnam had a smaller mean baseline volume, resulting in a faster volume reduction rate, achieving a VRR of 91.33% at the 12 month follow-up; meanwhile, in Taiwan the 12 month follow-up showed a VRR of 83.32% and further achieved a VRR of 95.23% at the 24 month follow-up. Furthermore, Taiwan showed a lower complete disappearance rate (53.6%) at the 12 month follow-up compared to Vietnam (86.7%), although a similar complete disappearance rate at the 24 month follow-up (Taiwan 98.3% vs. Vietnam 100%). This result could be attributed to the relatively larger ablation zone required as a safety margin for the larger tumors in Taiwan, which subsequently influenced the VRR and complete disappearance rate.

We also observed a statistical difference in the mean longest diameter between Taiwan and Vietnam, with the measurement being close to 5 mm in Vietnam and approximately 2 mm larger in Taiwan. Of note, recent studies have suggested that PTMC with a tumor size > 5 mm could be a possible predictive factor of tumor recurrence [33] and lymph node metastasis [34].

Additionally, we conducted a post hoc analysis to compare treatment outcomes between high-volume centers (≥ 50 cases) and low-volume centers (< 50 cases). The findings support the feasibility of achieving consistent treatment outcomes across centers of varying case volumes, provided that procedures are performed by trained operators following standardized protocols, even if early post-treatment response patterns may differ.

We also noted a statistical difference in the mean patient age between Taiwan and Vietnam. Patient age at the time of a thyroid cancer diagnosis is considered a crucial prognostic factor for survival, with an age cut-off of 55 years noted in the American Joint Committee on Cancer (AJCC) staging system for differentiated thyroid cancer [35]. Interestingly, recent studies have proposed new models related to the natural history of thyroid cancer, further dividing thyroid cancer into self-limiting and lethal cancers based on the age of 60 [36]. However, the epidemiology and prognosis of thyroid carcinomas in relation to age remain widely debated. We hypothesize that differences in terms of geogenomics, lifestyles, environmental exposures, and healthcare systems between Taiwan and Vietnam may contribute to the age differences at diagnosis, although further investigation is required to clarify the causes of these differences.

Although there is currently no formal population-based screening program for thyroid cancer in either Taiwan or Vietnam, routine thyroid US examinations are commonly included in elective health check-up packages offered by both commercial and hospital institutions, supported by the widespread availability and affordability of sonography. Local clinicians have noted that the frequent use of neck US has contributed to increased detection of small thyroid nodules, leading to a rise in US-guided FNAs and a corresponding increase in thyroid cancer diagnoses over the past 2 decades [3, 4].

While the 2015 ATA guidelines discourage routine FNA for TR-5 nodules < 1 cm, clinicians in our institutions may still perform FNA in selected cases when patients express anxiety or have a relevant family history. This approach aligns with a broader regional consensus in Asia, where guidelines recommend FNA for nodules measuring ≥ 5 mm when high-suspicion features are present on US. For example, the Korean Society of Thyroid Radiology recommends biopsy of category 4 or 5 K-TIRADS nodules ≥ 5 mm in the presence of additional risk factors such as suspicious lymph nodes or extrathyroidal extension [37].

Although early detection of PTMC enables minimally invasive treatment options, this de facto screening practice raises important concerns regarding potential overdiagnosis and the appropriate use of healthcare resources, particularly for PTMCs measuring 5–7 mm. We acknowledge this ongoing debate and would like to emphasize the importance of shared decision-making and individualized risk stratification to balance the benefits of early intervention with the risk of overtreatment.

This study has several limitations. First, the relatively short follow-up duration is a major limitation of our study, with 70.4% of tumors monitored for over 12 months. While this allowed for meaningful assessment of short- to mid-term outcomes, an extended follow-up report is necessary to allow comparison with active surveillance studies, which reported a progression rate of 3%–5% at 5 years [38].

Second, as a retrospective study, it has inherent limitations including potential selection bias and lack of randomization. Third, the study enrolled a relatively small number of patients. There was also heterogeneity in terms of the number of tumors treated at the different medical centers (Table 1). Fourth, although the general method of RFA was consistent, it must be noted that the RFA procedures in this study were performed by 5 different operators, which may have led to procedural differences.

## 5. Conclusion

We herein demonstrate that US-guided RFA is an established and reliable treatment option which is safe and effective for patients with PTMC, regardless of differences in terms of the individual operator, medical environment, and sociosystemic setting between Taiwan and Vietnam. The low complication rate and high success rate in achieving complete tumor ablation make RFA a promising minimally invasive alternative to surgery for PTMC.

## Figures and Tables

**Table 1:** Characteristics of patients and tumors.

	Taiwan					Vietnam			Total	*p*
Center (no.)	1	2	3	4	Total	5	6	Total		

Patients (*n*)	44	18	6	6	74	127	5	132	206	
Age (year)	51.36 ± 12.31	44.00 ± 11.47	50.4 ± 11.52	48.67 ± 23.23	49.28 ± 13.25	43.02 ± 11.71	35.91 ± 7.92	42.33 ± 11.44	44.82 ± 12.54	< 0.001
Gender (M/F)	12/32	4/14	2/4	1/5	19/55	23/104	2/3	25/107	44/162	0.399

Tumor characteristics										
Number (single/multiple^+^)	50 (38/12)	18 (18/0)	6 (6/0)	6 (6/0)	80 (68/12)	138 (116/22)	6 (4/2)	144 (120/24)	224 (188/36)	0.745
Longest diameter (mm)	7.54 ± 2.23	7.67 ± 1.75	6.44 ± 3.78	6.87 ± 1.65	7.44 ± 2.22	5.54 ± 1.58	7.23 ± 1.88	5.61 ± 1.62	6.26 ± 2.05	< 0.001
Baseline volume (mL)	0.18 ± 0.20	0.19 ± 0.11	0.18 ± 0.13	0.12 ± 0.06	0.18 ± 0.17	0.07 ± 0.06	0.13 ± 0.11	0.07 ± 0.06	0.11 ± 0.12	< 0.001
Location (*n*, (%))										0.090
Left	19 (38.0%)	10 (55.6%)	2 (33.3%)	2 (33.3%)	33 (41.3%)	44 (31.9%)	3 (50.0%)	47 (32.6%)	80 (35.7%)	
Right	28 (56.0%)	8 (44.4%)	4 (66.7%)	4 (33.3%)	44 (55.0%)	77 (55.8%)	3 (50.0%)	80 (55.6%)	124 (55.4%)	
Isthmus	3 (6.0%)	0	0	0	3 (3.7%)	17 (12.3%)	0	17 (11.8%)	20 (8.9%)	

Complications (*n*, (%))	0	0	0	0	0	3 (2.4%)	1 (20%)	0	4 (1.9%)	0.092
Repeat FNA at 6 months	31 (70.5%)	13 (72.2%)	4 (66.7%)	4 (66.7%)	51 (68.9%)	81 (63.8%)	3 (60.0%)	84 (63.6%)	135 (65.9%)	0.148
Disease progression (*n*, (%))	0	1 (5.6%)	0	0	0	0	0	0	1 (0.5%)	0.179

*Note:* Values are shown as mean ± standard deviation unless otherwise indicated. *p*: Calculated using data of Taiwan (total) and Vietnam (total).

Abbreviation: FNA = fine needle aspiration.

^+^All patients with multiple tumors had two papillary thyroid microcarcinoma (PTMC) tumors.

**Table 2:** Mean volume of tumors and VRR at each follow-up after ablation based on countries.

Country	Taiwan	*p*	Vietnam	*p*	Total	*p*	*p* ^#^	Group^∗^ time effect *p* value
Tumor volume (mean ± SD, ml)								0.193
Baseline	0.18 ± 0.17		0.07 ± 0.06		0.11 ± 0.12		< 0.001	
1 month	0.42 ± 0.32	< 0.001	0.41 ± 0.22	< 0.001	0.41 ± 0.26	< 0.001	0.370	
3 months	0.20 ± 0.19	< 0.001	0.14 ± 0.12	< 0.001	0.17 ± 0.15	< 0.001	0.170	
6 months	0.12 ± 0.19	< 0.001	0.04 ± 0.06	< 0.001	0.07 ± 0.13	< 0.001	< 0.001	
12 months	0.03 ± 0.08	< 0.001	0.01 ± 0.03	< 0.001	0.02 ± 0.06	< 0.001	< 0.001	
24 months	0.01 ± 0.03	< 0.001	0 ± 0	0.317	0.01 ± 0.03	< 0.001	0.159	
Time effect *p*-value	< 0.001		< 0.001		< 0.001			

Volume reduction ratio (mean ± SD, %)								< 0.001
1 month	−374.42 ± 639.23		−829.24 ± 906.28		−674.85 ± 851.66		< 0.001	
3 months	−81.20 ± 206.21	< 0.001	−210.51 ± 364.02	< 0.001	−165.46 ± 323.47	< 0.001	0.001	
6 months	4.52 ± 147.01	< 0.001	23.63 ± 90.67	< 0.001	16.80 ± 114.07	< 0.001	0.740	
12 months	83.32 ± 36.04	< 0.001	91.33 ± 43.60	< 0.001	87.58 ± 40.29	< 0.001	< 0.001	
24 months	95.23 ± 14.74	< 0.001	100 ± 0	0.317	96.18 ± 13.29	< 0.001	0.159	
Time effect *p*-value	< 0.001		0.003		< 0.001			

Cumulative disappearance (*n*, (%))								
1 month	0		0		0			
3 months	0		2 (1.3%)		2 (0.9%)			
6 months	8 (10.0%)		23 (16.0%)		31 (13.8%)			
12 months	37 (53.6%)		72 (86.7%)		109 (71.7%)			
24 months	58 (98.3%)		73 (100%)		131 (99.2%)			

*Note:* Values are shown as mean ± standard deviation unless otherwise indicated. *p*: Calculated using data of each follow-up time and the previous follow-up time. *p*^#^: calculated using data of Taiwan and Vietnam at the same follow-up time.

**Table 3:** Treatment outcomes based on center volume.

	High-volume centers	Low-volume centers	*p*
Centers (no.)	1, 5, 6	2, 3, 4	
*n*	194	30	

Baseline tumor volume (mean ± SD, mL)	0.10 ± 0.12	0.17 ± 0.11	0.003

Volume reduction ratio (mean ± SD, %)			
6 months	8.44 ± 120.20	70.91 ± 19.86	0.005
12 months	86.91 ± 43.72	91.12 ± 9.99	0.648
24 months	96.27 ± 14.22	95.75 ± 7.13	0.921

Cumulative disappearance (*n*, (%))			
12 months	99 (71.7%)	10 (71.4%)	0.834
24 months	114 (100%)	17 (94.4%)	0.458

Complication rate (%)	5 (2.6%)	0 (0.0%)	0.374

*Note:* Values are shown as mean ± standard deviation unless otherwise indicated. *p*: Calculated using data of high-volume centers and low-volume centers.

## Data Availability

Some or all datasets generated during and/or analyzed during the current study are not publicly available but are available from the corresponding authors on reasonable request.
